# Effect of Refined Edible Oils on Neurodegenerative Disorders

**DOI:** 10.34172/apb.2023.060

**Published:** 2022-11-02

**Authors:** Fataneh Hashempour-Baltork, Parastou Farshi, Adel Mirza Alizadeh, Sevda Eskandarzadeh, Solmaz Abedinzadeh, Sodeif Azadmard-Damirchi, Mohammadali Torbati

**Affiliations:** ^1^Halal Research Center of IRI, Iran Food and Drug Administration, Ministry of Health and Medical Education, Tehran, Iran.; ^2^Food Science Institute, Kansas State University, Manhattan, KS, USA.; ^3^Department of Food Science and Technology, National Nutrition and Food Technology Research Institute, Faculty of Nutrition Science and Food Technology, Shahid Beheshti University of Medical Sciences, Tehran, Iran.; ^4^Department of Nutrition, School of Public Health, Iran University of Medical Sciences, Tehran, Iran.; ^5^Department of Food Science and Technology, Faculty of Nutrition, Tabriz University of Medical Sciences, Tabriz, Iran.; ^6^Department of Food Science and Technology, Faculty of Agriculture, University of Tabriz, Tabriz, Iran.

**Keywords:** Vegetable oil, Refining process, Virgin oil, Health effect, Neurodegenerative diseases

## Abstract

Neurodegenerative diseases are comprise a prominent class of neurological diseases. Generally, neurodegenerative diseases cannot be cured, and the available treatments can only regulate the symptoms or delay the disease progression. Among the several factors which could clarify the possible pathogenesis of neurodegenerative diseases, next to aging as the main risk, the dietary related diseases are the most important. Vegetable oils, which are composed of triacyclglycerols as the main components and several other components in a trace amount, are the main part of our diet. This review aims to study the effect of refined or unrefined vegetable oil consumption as a preventive or aiding strategy to slow or halt the progression of neurodegenerative diseases. In the refining process, owing to the chemical materials or severe temperatures of the refining process, removal of the desirable minor components is sometimes unavoidable and thus a worrisome issue affecting physical and neurological health.

## Introduction

 Neurodegenerative diseases refer to various chronic disorders related to the progressive perceptual motor and sensory dysfunction, which leads to behavioral and cognitive impairments. In these pathologies, within different areas of the brain, the selective neuronal cell loss appears.^1^ Globally, more than 10 million people are diagnosed with neurological diseases yearly, which is expected to grow by 20%–25% over the next decade.^2^ Recently, a growing interest has been arising in identifying the risk factors and mechanisms which lead to the complex etiopathogenesis of neurodegenerative diseases including vascular, genetic, metabolic, and lifestyle-related factors that often exist together and interact with each other.^3,4^ Among the several factors which could clarify the possible pathogenesis of neurodegenerative diseases, next to aging as the main risk, the dietary related diseases including cerebrovascular diseases, inflammation, and diabetes, are of the most important.^5^

 Oils have important roles in food formulations and can make a significant contribution to diet and health.^6,7^ Vegetable oils are consumed in both virgin and refined types, though there has been a growing interest in virgin oils as a functional food.^7,8^ In the refining process, the elimination of the unwanted components while maintaining the essential elements of the oil is critical. However, owing to the severe chemicals and the high temperature of the refining process, removal of desirable components is sometimes unavoidable, thereby a worrisome issue for health. According to the literature, neutralization, bleaching and deodorization lead to a reduction in the contents of γ-oryzanol, squalene, tocopherol/tocotrienol and phytosterols, phenol and β-carotene.^7^ However, these bioactive components and other antioxidants play a significant role in the reduction of the oxidative stress associated with chronic diseases, neurodegenerative diseases, cancers, diabetes, and heart diseases.^7,8^ Additionally, the oxidation of lipids and their related substances found in the refined oils and oil products, which are toxic and harmful, could affect the quality and function of the products. The type of the consumed fat has a principal role in the health of individuals, while higher consumption of trans fatty acids is known to have unfavorable health effects.^9^ Nonetheless, there is still a gap of knowledge in choosing the best type of edible oils. Due to the importance of this issue, the present paper presents an overview of the effects of refined and unrefined oils consumption on neurological aspects.

## Vegetable oil refining

**Figure 1:**
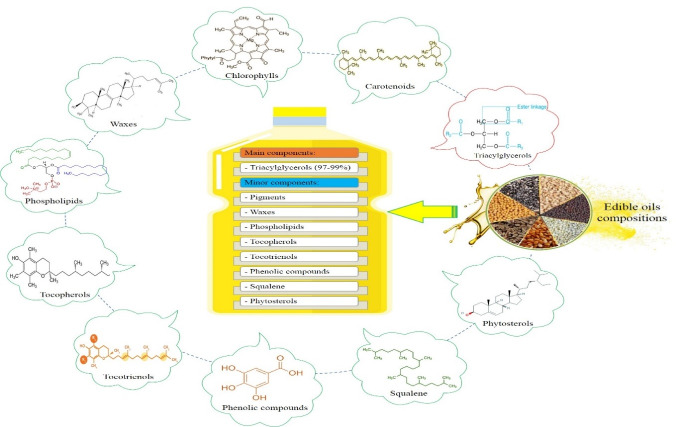
Edible oil composition.

 Vegetable oils are composed mainly of triacylglycerols (97-99%) and some minor components (Figure 1). Minor components are low in quantity but have many different technological and nutritional effects which make vegetable oils different from many aspects.^8^ It should be noted that there are two classes of minor components, namely useful and harmful from technological and nutritional points of view. Generally, a big portion of the minor components has positive effects; however, there are a few minor components with adverse effects such as gossypol, peroxides, free fatty acids, etc., which should be removed from vegetable oils.^8,9^

**Figure 2:**
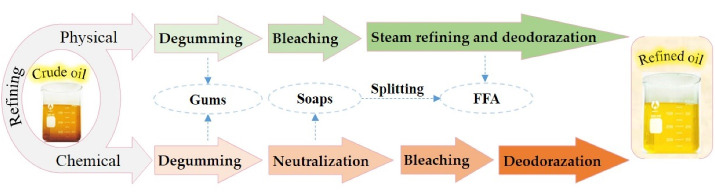
physical and chemical refining steps.

 Refining is used to remove unwanted components from vegetable oils. Refining can be done in different ways, mainly including chemical refining and physical refining. Refining steps are presented in Figure 2. The main difference between the chemical and physical refining is the way of free fatty acid removal. Free fatty acids are removed through neutralization by alkali and deodorization (steam distillation) in chemical and physical refining, respectively.^7^

## Oxidative stress cell damages and inflammation

 Inflammation is one of the main connectors, linking vascular neurodegeneration and abnormalities. Certainly, inflammation, particularly of the endothelium, can initiate a broad spectrum of age-related neurodegenerative diseases and has a role in their progression. Also, it has been demonstrated that it can affect the expression of brain-derived neurotrophic factor.^10^ Neuroinflammation is a main factor in both chronic and acute conditions.^11^ Two nuclear factors such as (erythroid-derived 2)-like 2 (Nrf2) and κB (NF-κB) are interrelated master regulators of cellular responses to oxidative stress and inflammation, respectively.^12^ Recently, many studies have indicated that dysfunctions in redox homeostasis are common mechanisms in metabolic and neurological diseases.^13^

 It is clear that constant consumption of the traditional Mediterranean diet, which includes extra virgin olive oil (EVOO) and nuts containing many components with positive health effects, not only hinders neuroinflammation and oxidative stress but also improves the immune function. The antioxidant activities of olive oil are attributed to the presence of tyrosol, oleuropein, and hydroxytyrosol. These are the major components in VOO, and are known to have neuroprotective activities, as well as antioxidant and anti-inflammatory properties.^14^ Moreover, as it was mentioned, the conversion of oleic acid into nitro‐oleic acid prevents neuroinflammation by blocking the activation of NF‐κB and prevention of oxidative stress through the stimulation of the Nrf2 transcription factor. Furthermore, neuroinflammation in neurological disorders may stimulate pain, which can be caused by the activation of primitive nociceptive sensory and somatosensory nerves. EVOO components have been shown to activate the TRPA1 receptors (TRP ion channel family of receptors). These processes, besides the synthesis of nitro‐oleic acid, can reduce the pain intensity by stimulating nociceptive neurons through a TRPA1 receptor‐mediated process. Evidence shows that nitro‐oleic acid can act as an endogenous peroxisome proliferator‐ activated receptor gamma (PPARγ) ligand, which has vascular protective effects and can downregulate the NF‐κB proinflammatory genes’ expression; also, it is able to upregulate the Nrf2, which is an antioxidant transcription factor.^14^

 Free radicals are naturally produced compounds in human, animal and even plant organs and can cause several cell damages. Oxidative stress, which occurs due to free radicals, can lead to inflammation which can be lessened by antioxidant-protective system. The differences between the effect of refined olive oil (ROO) and EVOO against oxidative stress was attributed to the higher phenolic compounds present in EVOO, which resulted in higher protection against the oxidative stress caused by this oil.^8^

 Melatonin is a phytochemical which has strong antioxidant and radical scavenging properties as well as anti-inflammatory and immunomodulatory activities. Melatonin is shown to be higher in EVOO, which is one of the major constituents of Mediterranean diet in comparison to ROO.^15^

 Besides, the anti-inflammatory effect of virgin coconut oil (VCO) was shown in a study investigating its suppressive effect on the oxidative stress caused by methotrexate drug, which is used in cancer patients receiving chemotherapy, and the results showed that VCO is a potential compound for regulating the neurotoxicity of methotrexate in cancer patients.^16^

 On the other hand, there are some compounds found in oxidized oils, such as alkyl hydroperoxides, which can cause oxidative damage to different body tissues. The canolol from crude rapeseed, which is highly reduced in refined oil, is a good scavenger of alkyl peroxyl radicals. The radical scavenging characteristic of canolol is higher than that of other antioxidants such as quercetin, vitamin C, β-carotene, and α-tocopherol.^17^ The OOH radical scavenging ability of canolol has also been shown, which makes it a potential compound to prevent oxidative stress.^18^ In a study investigating the beneficial clinical effects of cold-pressed primrose oil in comparison to the refined one, it was revealed that cold pressed oil has higher free radical scavenging properties because of its higher amounts of triterpenes.^19^

 In a study, palm oil carotenes and their tocotrienol-rich fractions were shown to have down regulating effects on pro-inflammatory markers such as interleukin (IL)-6, IL-β, tumor necrosis factor (TNF), plasma C-reactive protein (CRP) and plasma immunoglobulin E (Ig E), while increasing the IL-4 and 3.^20^ The protective effect of red palm oil and its potential mechanisms of the neuroprotective actions were demonstrated in a comprehensive review paper.^21^ Oxidative stress and inflammatory studies showed that generally, virgin or crude oils containing high amounts of bioactive micronutrients such as antioxidant compounds have higher antioxidant and anti-inflammatory activities. Table 1 indicates the effect of different virgin oils on inflammation markers

**Table 1:** Effects of different virgin oils on inflammation markers

**Type of oil**	**In vivo/In vitro Model**	**Consumption dose**	**Effect**	**References**
EVOO phenolic extracts	Caco-2 cells	5–25 μg/mL	Inhibition of p38 and ERK1/2 activation and of IĸBα degradation	^22^
EVOO	Immune-mediated disease patient	50 mL/d	TNF-α and CRP indicated significantly reduction after 20 days	^23^
VCO	Rat	5 and 15% (w/w), 14 days	reducing oxidative stress and pro-inflammatory responses in supplementation by methotrexate (reduction in SOD, CAT, GPx and GSH)	^24^
EVOO phenolic extracts	Macrophages J774 A.1 stimulated with LPS	50–150 μg/mL	Inhibition of NO over-production and of COX-2 and iNOS expression	^25^
VCO	THP-1 cells (Human monocytes)	200 μg/mL	VCO inhibited TNF-α (62.34 ± 3.2 %), IFN-γ (42.66 ± 2.9 %), IL-6 (52.07 ± 2.0 %), IL-8 (53.98 ± 1.8 %) and IL-5 (51.57 ± 2.6 %)	^26^
EVOO phenolic extracts (Oleocanthal)	LPS-activated human primary osteoarthritis chondrocytes	1–5 μM	Inhibition of NO over-production following inhibition of iNOS expression through MAPK modulation	^27^
Red palm oil	Brown Norway rats	30 mg/kg body weight	Downregulated pro-inflammatory markers (IL-β, IL-6, TNF-α), coincident with anti-inflammatory marker IL-4 and IL-13 upregulation. Treatment significantly reduced asthmatic rat plasma CRP and IgE, signifying improved systemic inflammation.	^20^
Sesame seed oil essential oil	spleen cells of mice	0.01–100.0 μg/mL	the sesame ingredients reduced the release of IFN-γ and increased secretion of IL-4 from lymphocytes. Macrophages viability was not affected and production of NO, TNF-α, and IL-1β were inhibited using sesame essential oil and sesamol.	^28^

ERK1/2: extracellularly-regulated kinase-1 and -2; SOD: superoxide dismutase; CAT: catalase; GPx: glutathione peroxidase; GSH: glutathione; NO: nitric oxide; COX-2: cyclooxygenase-2; iNOS: inducible nitric oxide synthase; IFN-γ: Interferon gamma; MAPK: Mitogen-activated protein kinase 1; EVOO, extra virgin olive oil; VCO, virgin coconut oil.

## Neurological disorders

 Diseases that destroy or impair the function of neurons in the spinal cord and brain are identified as “neurological disorders”. The cause of these diseases can be neurochemical, electrophysiological, or structural abnormalities in the spinal cord, nerves, and brain, which results in neurodegeneration leading to poor coordination, muscle weakness, paralysis, memory loss, confusion, seizures, and pain.^29^ In neurological disorders, neurodegeneration is a multifactorial and complex process followed by the onset of neuroinflammation, initiation of oxidative stress, misfolding, oligomerization of proteins, dysregulation of calcium, DNA damage, mitochondrial dysfunction, deficits in axonal transport, and irregular RNA processing.^1^ Neurological disorders include neurodegenerative, neuropsychiatric, and neurotraumatic diseases. In this study, the main neurodegenerative diseases in association with refined or virgin oils were considered as below:

###  Alzheimer

 Alzheimer’s disease (AD) is a neurodegenerative defect and the prominent cause of dementia in the aging population. Despite the unclear cause of AD, the principal features usually detected in the brains of AD patients include hyperphosphorylated tau protein aggregation, amyloid-β plaques protein deposition, neuropil threads, neuronal and synapsis loss, abnormal neurites, microglial activation, astrogliosis, disordered blood−brain barrier, and cerebral amyloid angiopathy.^30^ Several neuroinflammations, such as activation of LRR, PYD domain-containing protein 3 (NLRP3), and NACHT, inflammation in microglia and boosted IL-1β levels can also occur in AD.^30^ Besides the core pathological features of AD, there is more evidence on the increased oxidative stress, which contributes to the progression of this complex disease.^31^

 VCO was able to reduce NLRP3 expression and oxidative stress, which had been stimulated by Amyloid-β and the high fat diet in a rat model of AD, which confirmed its neuroprotective effect.^32^ It has been found out that ketones, which are formed by the conversion of medium-chain-triglycerides in specific foods, are energy sources for brain and coconut oil is a rich source of these compounds.^33^ The levels of medium-chain-triglycerides are the same in VCO and RCO; thus, their effect in increasing ketone form of beta-hydroxybutyrate (beta OHB) in Alzheimer patients would be the same.^34^ Moreover, the neuroprotective antioxidant activities of coconut oil have been attributed to its polyphenolic content, which is present in higher concentrations in virgin oils (such as caffeic acid, gallic acid, quercetin, myricetin glycoside catechin, methyl catechin, dihydrokaempferol, ferulic acids, and p-coumaric acid).^35^

 Based on the literature, there is enough evidence on the link between the Mediterranean diet of which EVOO is the main constituent, and lower risk of AD. Studies have shown that adding EVOO addition to the AD model mice diet before the onset of pathology resulted in the restoration of blood−brain barrier function, reduction of the levels of total brain tau, and amyloid-β; it also improved the cognitive behavior in the mice.^30,36^ Intake of oleocanthal-rich EVOO provides protection against the AD progression even at the progressive stages of it by decreasing the activation of NLRP3 inflammasome and provoking autophagy via AMP-activated protein kinase/Unc-51-like and autophagy activating kinase 1 pathway (AMPK−ULK1).^30^ The main phenolic compounds in perilla oil (luteolin and apigenin) in addition to rich α-linolenic acid, which are removed or decreased to very low amounts through refining, were shown to improve neuropsychological disorders such as AD by activating the monoamine transporter in the tested patients.^37,38^ According to the reports, virgin edible oils have different useful neurodegenerative effects by reducing the levels of total tau protein fractions, and amyloid-β peptide of brain, as well as improving the blood−brain barrier function,

###  Parkinson

 In Parkinson’s disease (PD), which is a neurodegenerative disorder, dopamine-producing nerve cells start to die off. L-dopa is considered as the most effective compound for treating PD. However, there are some limitations for long-term use of this compound. VCO has considerable protective effect against detrimental activities of L-dopa, such as motor response oscillation and dyskinesia, which can be related to polyphenols, medium-chain-triglycerides, and other anti-inflammatory compounds present in this oil.^39^ To confirm the neuroprotective property of VCO, an experiment was conducted investigating the effect of VCO on rats which were exposed to benzene inhalation. VCO was shown to be considerably effective in attenuating the adverse effects of benzene exposure, namely increased lipid peroxidation and lower glutathione (GSH) and dopamine levels (in brain tissues).^40^

###  Multiple sclerosis 

**Figure 3:**
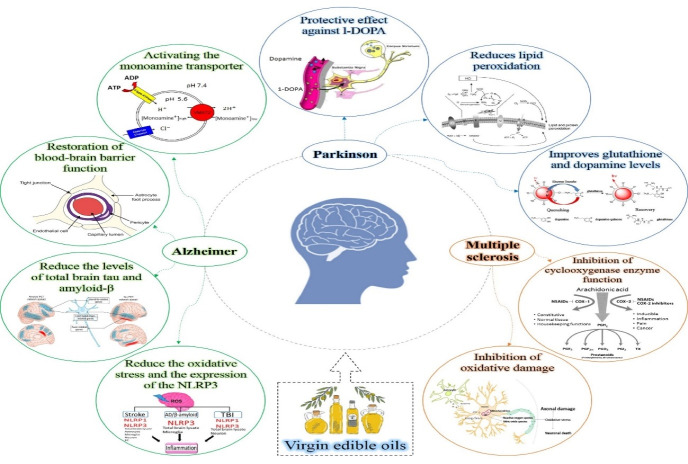
Effect and mechanism of virgin oil on neurodegenerative disorders

 Multiple sclerosis (MS) is a chronic inflammatory disease of the central nervous system. This inflammatory demyelinating disease may occur in response to a chronic viral infection or as a reflection of a dysfunctional immune system.^41^ Oleocanthal, a major phenolic compound of EVOO was revealed to prevent the cyclooxygenase enzymes that are involved in tumorigenesis and demyelination. Therefore, researchers hypothesized that the Mediterranean diet would also provide protection against MS.^42^ In a study on rats, it was shown that gastric administration of EVOO could decrease the oxidation degree of protein and lipid and enhance the GSH peroxidase, which makes it a suitable diet to provide protection against oxidative damage. Moreover, it decreased the levels of bacterial lipopolysaccharide and lipopolysaccharide-binding proteins, which are produced as a result of oxidative stress caused by autoimmune encephalomyelitis.^42^ In addition, there are several studies on the positive effects of omega 3 (n-3) fatty acids on MS and the oxidative damage caused by MS.^43,44^ However, the differences between the effects of crude and refined oils on MS seem to be highly dependent on the refining process and parameters. In a study on the effect of primrose oil on life quality and fatigue in the patients with MS, it was shown that including this oil in the diet of MS patients could significantly increase the cognitive function, happiness and general life satisfaction in them.^45^ This effect of primrose oil may be due to its γ‐linolenic acid content. Although refining the primrose oil decreases its micronutrients content, it does not significantly affect its fatty acid amounts.^19^ Based on the studies, it can be inferred that there is still need for further investigation on the effect of refined and crude oils on MS progression. Figure 3 presents the effect and mechanism of virgin oil on neurodegenerative disorders.

###  Other disorders 

 Gut microbiota consist of a group of live microorganisms inhabiting the digestive tract. The microbiota is necessary for an accurate body growth, as well as for developing immunity and nutrition. Research into the role of the gut microbiome in regulating the brain function has rapidly grown over the past 10 years. The enhancing preclinical and clinical evidence implicates the microbiome as a potentially fundamental susceptibility factor for neurological disorders such as AD, MS, PD, autism spectrum disorder, and stroke.^46^ The gut microbial composition changes according to diet and age, during infancy and throughout life. A study on the modulation of gut microbiota by Mediterranean diet showed that these compounds could regulate the gut microbial metabolism and composition, enhance the gut bacterial diversity and provide favorable effects.^47^

 Including olive oil phenolic compounds in the diet results in the reduction of *Firmicutes/Bacteroidetes *ratio and the increase in *Bacteroidetes*, *Bifidobacteria* and, in some cases, *Lactobacillus*. In general, they can exert atheroprotection and beneficial effects on obesity, and immune and cognitive disorders.^46,48^ Investigation into the effect of ROO and EVOO on intestinal microbiota of mice showed that polyphenols and other minor compounds in EVOO can have a part in preventing the undesirable bacteria (from *Helicobacteraceae, Spiroplasmataceae,* and *Desulfovibrionaceae* families). However, they could indirectly help to increase the two other families of bacteria (*Sutterellaceae*and *Erysipelotrichaceae*), the mechanism of which requires more considerations.^49^ Comparing the effect of ROO, EVOO, and butter on gut microbiota indicated the unique behavior of EVOO in changing the gut microbiota, which led to its desirable effects on the inhibition of metabolic syndrome and obesity.^50^ Literature emphasizes the role of phenolic compounds present in virgin oils, which are mostly degraded after refining, in regulating gut microbiota. Table 2 presents the studies conducted in association with the effect of refined and unrefined oils on neurological disorders.

**Table 2:** Effects of refined and unrefined oils on neurological disorder

**Disorder**	**Type of oil**	**Patient**	**Dose**	**Duration**	**Result**	**Effective compound**	**Ref**
AD	VCO	120 male Wistar rats (8 weeks old)	8% and 10%	0.5 weeks	Improvement in the hippocampus health and learning and memory and in AD and high fat diet model rats	Phenolic compounds	^32^
	Oleocanthal-rich EVOO in combination with donepezil	12 group of mice, each 6 females and 6 males (one month old)	0.7 g/kg/d	16 weeks	EVOO could change the amyloid precursor protein’s pathway processing to a nontoxic one; it causes a decrease in overall load of Aβ in brain which may be related to its ability to activate secretase-α and to inhibit the activity of secretase-β (enhances donepezil’s effect)	Oleocanthal and oleuropein in EVOO	^36^
	Refined and EVOO	2 groups of 7 female mice	0.714 (g/kg)/day, containing 476 (μg/kg)/day oleocanthal in the EVOO group.	3, 9, 12 months	Supplementation of Oleocanthal-rich extra virgin olive can slow down or stop the progression of AD	Phenolic compounds in EVOO, especially oleocanthal	^30^
PD	VCO	40 male adult Sprague Dawley rats (150−200 g)	1.42 mL/kg	4.2 weeks	Providing protection against harmful effects of L-dopa	Polyphenols and tocopherols	^39^
MS	EVOO	25 male dark Agouti rat (2 month old, 190-200 g)	10% of the calorie intake (in group fed with EVOO)	9.3 weeks	The oxidation degree of protein and lipid decreased and GPx increased, providing protection against oxidative stress	Antioxidant content	^42^
Gut microbiota	Refined and EVOO	35 male Swiss Webster mice in 4 groups with different diets	35% of total energy	12 weeks	EVOO diet resulted in lower levels of some of undesirable gut bacteria in comparison to ref­ined oil diet	Polyphenols in EVOO	^49^
	Hydroxytyrosol (main component of virgin olive oil)	28 male (3-week-old)	50 mg/Kg/d	8 weeks	This compound could alter the gut microbiota, thus good for treating obesity and insulin resistance	Hydroxytyrosol	^51^

MS, multiple sclerosis; EVOO, extra virgin olive oil; GPx: glutathione peroxidase; VCO, virgin coconut oil; PD, Parkinson’s disease; AD, Alzheimer’s disease.

## Conclusion

 Nowadays consumers prefer healthy, natural, and beneficial food products, such as cold pressed oils. Recently, more general attention is paid to cold pressing method which is considered to be the preferred method for the extraction of oils from oilseeds and fruits, due to its desirable properties e.g., being inexpensive and user-friendly, in comparison to other extraction methods. Cold pressing technique of oil extraction doesn’t involve heating or chemical treatment, which may retain higher amounts of minor bioactive compounds, such as natural antioxidants, tocopherols, phytosterols, phenolic compounds, squalene, phospholipids, pigments, and aroma and flavor compounds. Minor fractions play a significant role in determining the health and nutritional effects of edible oils. On the other hand, the role of the phenolic compounds presents in virgin oils, which are mostly degraded after refining, is emphasized in the regulation of gut microbiota, which can affect the mental and neurological characterization.

 Literature has reported many health effects related to crude oils, such as their beneficial effects on various diseases including neuro defects. Including EVOO, VCO, red palm oil and other virgin oils rather than refined types into daily diet can regulate metabolic and mental changes. In some neurodegenerative defects such as Alzheimer and Parkinson, virgin edible oils proved their priority by restoration of blood−brain barrier function, reducing the levels of total tau protein, and amyloid-β peptide of brain due to their beneficial bioactive micronutrients. But in relation to MS, it was shown that gastric administration of EVOO decreases the oxidation degree of proteins and lipids and enhances the GSH peroxidase, which makes it a suitable diet to provide protection against oxidative damage. Yet, it decreases the levels of bacterial lipopolysaccharides and lipopolysaccharide-binding proteins, which are produced as a result of oxidative stress caused by autoimmune encephalomyelitis. Thus, it can be concluded that there is still need for further investigation into the effect of refined and crude oils on MS progression.

## Acknowledgments

 This study has been supported by the Halal Research Center of IRI, Iran Food and Drug Administration, Ministry of Health and Medical Education, Tehran, Iran. As well, we gratefully acknowledge the assistance of Department of Food Science and Technology, Tabriz University of Medical Sciences and University of Tabriz.

## Competing Interests

 The authors declare that there is no conflict of interests.

## Ethical Approval

 Not applicable.
